# Task shifting and integration of HIV care into primary care in South Africa: The development and content of the streamlining tasks and roles to expand treatment and care for HIV (STRETCH) intervention

**DOI:** 10.1186/1748-5908-6-86

**Published:** 2011-08-02

**Authors:** Kerry E Uebel, Lara R Fairall, Dingie HCJ van Rensburg, Willie F Mollentze, Max O Bachmann, Simon Lewin, Merrick Zwarenstein, Christopher J Colvin, Daniella Georgeu, Pat Mayers, Gill M Faris, Carl Lombard, Eric D Bateman

**Affiliations:** 1 Knowledge Translation Unit, University of Cape Town Lung Institute, University of Cape Town, Cape Town, South Africa; 2 Department of Medicine, Faculty of Health Sciences, University of the Free State, Bloemfontein, South Africa; 3 Department of Medicine, University of Cape Town, Cape Town, South Africa; 4 Centre for Health Systems Research and Development, University of the Free State, Bloemfontein, South Africa; 5 School of Medicine Health Policy and Practice, University of East Anglia, Norwich, UK; 6 Norwegian Knowledge Centre for the Health Services, Oslo, Norway; 7 Health Systems Research Unit, Medical Research Council of South Africa, Cape Town, South Africa; 8 Sunnybrook Research Institute and Department of Health Policy, Management and Evaluation, University of Toronto, Toronto, Canada; 9 IHCAR, Karolinska Institute, Stockholm, Sweden; 10 Faculty of Medicine, University of Stellenbosch, Tygerberg, South Africa; 11 Centre for Infectious Disease Epidemiology and Research, School of Public Health and Family Medicine, University of Cape Town, Cape Town, South Africa; 12 Division of Nursing and Midwifery, School of Health and Rehabilitation Sciences, Faculty of Health Sciences, University of Cape Town, Cape Town, South Africa; 13 Biostatistics Unit, Medical Research Council, Cape Town, South Africa; 14 Department of Respiratory Medicine, University of Cape Town, Cape Town, South Africa; 15 University of Cape Town Lung Institute, University of Cape Town, Cape Town, South Africa

## Abstract

**Background:**

Task shifting and the integration of human immunodeficiency virus (HIV) care into primary care services have been identified as possible strategies for improving access to antiretroviral treatment (ART). This paper describes the development and content of an intervention involving these two strategies, as part of the Streamlining Tasks and Roles to Expand Treatment and Care for HIV (STRETCH) pragmatic randomised controlled trial.

**Methods: Developing the intervention:**

The intervention was developed following discussions with senior management, clinicians, and clinic staff. These discussions revealed that the establishment of separate antiretroviral treatment services for HIV had resulted in problems in accessing care due to the large number of patients at ART clinics. The intervention developed therefore combined the shifting from doctors to nurses of prescriptions of antiretrovirals (ARVs) for uncomplicated patients and the stepwise integration of HIV care into primary care services.

**Results: Components of the intervention:**

The intervention consisted of regulatory changes, training, and guidelines to support nurse ART prescription, local management teams, an implementation toolkit, and a flexible, phased introduction. Nurse supervisors were equipped to train intervention clinic nurses in ART prescription using outreach education and an integrated primary care guideline. Management teams were set up and a STRETCH coordinator was appointed to oversee the implementation process.

**Discussion:**

Three important processes were used in developing and implementing this intervention: active participation of clinic staff and local and provincial management, educational outreach to train nurses in intervention sites, and an external facilitator to support all stages of the intervention rollout.

The STRETCH trial is registered with Current Control Trials ISRCTN46836853.

## Background

South Africa has the largest human immunodeficiency virus (HIV) burden in the world, with an estimated 5.7 million infected people [1]. By the end of 2008, five years after the public sector antiretroviral treatment (ART) programme was launched, an estimated 700,500 people were accessing ART [2]. Although this represents an increase of 53% on the previous year, it constitutes only 40% of those estimated to be in need of ART [3]. Despite policy guidelines recommending that comprehensive HIV care be incorporated into existing primary care services [4], the initial public sector ART rollout in South Africa was implemented as a vertical (stand alone) programme with separate funding, facilities, staff, medical records, and reporting requirements [5]. There are several reasons to justify such an initial vertical approach to comprehensive HIV care, including the need for a rapid response in a weak health system and the need for highly skilled staff to implement a new, complex intervention [6,7]. There are, however, two equally powerful reasons for moving away from vertical HIV care programmes in high HIV-burden countries: that such vertical programmes will be unable to achieve universal ART access because of the sheer numbers of people needing treatment; and that they could draw away financial and human resources from already struggling public health systems in these countries [8,9].

In order to address these concerns, calls have been made to utilise the impetus of new financing, training, and infrastructural support, directed towards the acquired immunodeficiency syndrome (AIDS) epidemic, to strengthen broader health systems [10], and to incorporate current vertical ART programmes into these health systems--a strategy now termed the 'diagonal approach' [11]. Approaches to incorporating HIV care into general health systems include: the referral of patients stabilised on ART from ART clinics to primary care clinics where they could receive monthly supplies of treatment (sometimes referred to as 'down referral') [12,13]; task shifting of aspects of HIV care to lower cadres of healthcare workers [14,15]; setting up nurse-driven HIV care programmes [16]; and integration of HIV care into primary care services [17-19].

These types of interventions are complex, and there are two important research questions that need to be answered, particularly in low- and middle-income countries [20]: What should be the components of these interventions [21-23]? And are these interventions effective in improving access to ART? This article addresses the first question--it describes the content of the STRETCH (Streamlining Tasks and Roles to Expand Treatment and Care for HIV) intervention, including its components, the processes of change used, the conditions in the control clinics, and links to manuals used in the intervention, as suggested in the WIDER recommendations (Workgroup for Intervention Development and Evaluation Research) [24]. The development of the intervention was based on the educational outreach model and our practical experience of engaging with the Free State Department of Health in implementing an earlier nurse training programme called PALSA PLUS (Practical Approach to Lung Health and HIV/AIDS) in the Free State [25-27]. The second question is being addressed through a pragmatic cluster randomised controlled trial of the effects of the STRETCH intervention on access to ART conducted in 31 ART clinics randomised in nine strata in the Free State province [28]. This description will supplement the forthcoming trial results.

### Context and setting: the Free State public sector ART rollout

The Free State, with a population of 2.8 million [29], has an estimated HIV prevalence of 18.5% among 15 to 49 year olds [30]. The province comprises five districts, divided into 20 local areas, with primary care services offered at 222 nurse-led clinics. The public sector ART rollout commenced in mid-2004 in designated nurse-led ART assessment sites situated in selected primary care clinics. Table 1 summarises the organisation of HIV care in health facilities in the initial rollout. Patients diagnosed as HIV positive in primary care clinics and hospitals are referred to ART assessment sites for further clinical care and assessment of eligibility for ART. Those eligible for ART receive drug readiness training and are then referred to ART treatment sites in local hospitals for initiation of treatment and for three- to six-month reviews of ART prescriptions by a doctor. National regulations require that antiretrovirals (ARVs) be dispensed by or under the direct supervision of a pharmacist. Where assessment sites do not have pharmacists, ARVs have to be dispensed at treatment sites into patient-named packets and transferred to assessment sites where nurses issue them monthly to patients. In some remote areas, assessment and treatment site functions were conducted by combined sites with the support of visiting doctors.

**Table 1:** Responsibilities for provision of aspects of HIV care at different facilities in the initial ART rollout compared with responsibilities for sites in the STRETCH trial

Type of facility	Responsibilities for HIV care in initial ART Rollout	Responsibilities for HIV care for sites in the STRETCH trial
Primary care services	• Voluntary counselling and testing	• Voluntary counselling and testing • Initial CD4 count • Routine HIV care (repeat CD4 counts, clinical staging and TB screening) for patients not requiring ART • Drug readiness training • Baseline bloods • Monthly ART follow-up and issuing of ARVs (after first six months for stable patients)

ART assessment sites	• Initial CD4 count • Routine HIV care (repeat CD4 counts, clinical staging and TB screening) for patients not requiring ART • Refer patients eligible for ART (Stage IV AIDS or CD4 <200 cells/mm3) to doctor at treatment site • Drug readiness training • Baseline bloods • Monthly ART follow-up and issuing of ARVs	• Initiate uncomplicated patients on ART • Monthly ART follow-up and issuing of ARVs for first six months • Six monthly review and repeat ART prescription for stable patients • Refer complicated patients for initiation and repeat of ART prescription to doctor at treatment site

ART treatment sites	• Initiation of patients on ART • Monthly review first three months • Six monthly review and repeat ART prescription	• Initiation of complicated patients on ART • Monthly review first three months of complicated patients • Six monthly review and repeat ART prescription for complicated patients

In the first three years of the rollout, achievements included: good patient outcomes amongst patients receiving ART [31,32], a reliable supply of drugs and other medical supplies, and increases in nurse posts [33]. These successes were tempered by high mortality rates among patients waiting for ART [31], increased vacancies in primary care services [34], and high levels of burnout among ART and primary care nurses [35]. Despite opening 57 ART sites, coverage by the end of 2007 remained disappointingly low. Only 25% of new patients estimated to be in need of ART that year were started on treatment [36].

In late 2008, while the STRETCH trial was ongoing, the Free State ART programme was forced to implement a three-month moratorium on selected adult ART initiations to ensure uninterrupted drug supplies for those already on treatment. This moratorium was due in part to chronic underfunding of the ART programme in all provinces, and resulted in a major review and increase in funds for the national ART programme. In early 2010, before the STRETCH trial was completed, the South African government commenced implementation of its accelerated AIDS plan in all provinces. This plan includes nurse prescription of ART and integration of ART into all primary care clinics in an attempt to rapidly scale-up ART access [37]

### Developing the intervention

In 2005, Free State Department of Health managers expressed their concern about high mortality rates among patients waiting for ART, and about the dependence of the programme on doctors, who are in short supply, for ART prescription. Working in the Free State, the Knowledge Translation Unit of the University of Cape Town Lung Institute had piloted and evaluated a training programme for nurses in the use of integrated primary care guidelines covering the management of respiratory diseases and HIV--the PALSA PLUS initiative [25-27,38,39]. The provincial department thus requested that nurse prescription of ART be included in the PALSA PLUS guidelines, and that training be rolled out in the province. Because of widespread ambivalence about the ability of nurses to take on the clinical responsibility for ART prescription and the absence of clear national policy, it was decided to pilot the intervention and monitor its outcomes as a pragmatic randomised controlled trial in the province's ART clinics. Meetings were then held over eighteen months between researchers, managers, senior clinicians, and clinic staff to develop the intervention.

### Meetings with senior managers and clinicians

In initial meetings with senior managers and clinicians from the ART programme, it was established that delays in people accessing ART were caused not only by the shortage of doctors but also the high caseload of ART nurses at ART assessment sites that were managing growing numbers of patients on ART as well as those not yet eligible for ART. The intervention was therefore designed to be a more complex task-shifting intervention with two main components: shifting ART prescription from doctors to ART nurses and shifting routine HIV care for patients not yet eligible for ART (pre-ART care), from ART nurses to primary care nurses at ART assessment sites.

### Meetings with middle managers

Workshops were then held with district and local area managers to further develop the intervention. Managers expressed concern about the ability of nurses to assume these new clinical responsibilities and about how to implement the reorganisation of care required for this type of complex health intervention. It was agreed that in addition to providing nurse training, the intervention would be implemented in phases, and detailed descriptions of the task and role changes needed at intervention clinics in each phase would be included in an implementation 'toolkit' to be developed by the researchers.

### Meetings with clinic staff

To obtain feedback from clinic staff on the proposed intervention, the STRETCH coordinator (KU) visited all 31 nurse-led ART assessment clinics selected for the trial and held meetings with staff members. The staff raised a number of problems with functioning of the ART sites that were resulting in difficulties for patients accessing ART. These difficulties included increasing workload, drug transport and storage problems resulting from hospital-based ART dispensing, transport problems for patients, and lack of basic communication infrastructure such as telephones and fax machines (see Table 2). ART nurses were also struggling to cope with providing care for the growing numbers of patients accessing ART as well as those not yet eligible for ART. In one local area where primary care clinics did not offer HIV testing, ART staff had to provide this service too. However, in other districts, increasing workload had already prompted ART sites to integrate pre-ART care into the work of the surrounding primary care clinics. In one district, ART sites were already discussing the integration of drug readiness training, for patients eligible for ART, into primary care services.

**Table 2:** Problems in delivery of care at ART sites, as identified in initial clinic meetings

Operational issues	• Increasing workload as patients on ART were required to attend monthly to obtain supplies of ARVs • Staff shortages and delays in filling vacant post in the ART programme • Antagonism of primary care nurses toward ART nurses on account of their different post structures and remuneration leading to refusal to assist (some clinics) • Long delays in taking of CD4 counts because of lack of capacity in primary care services in some areas to perform voluntary counselling and testing and CD4 counts • Lack of integration of primary care services for patients on ART leading to multiple visits to healthcare facilities
Drug supply issues	• Shortage of pharmacists and pharmacy assistants • ARVs classified as hospital level medication which could only be dispensed by pharmacist • Shortage of transport to deliver dispensed ARVs to assessment sites • Lack of storage space and systems for locating individual patient's dispensed ARVs at assessment sites • Difficulty looking for individual patient's pack of dispensed ARVs • Differing availability of cotrimoxazole and fluconazole at ART service points

Transport issues	• Patients unable to afford taxi fares to attend treatment sites for doctor's assessment • Regular clinic transport systems becoming overwhelmed by increasing numbers of ART patients needing to go to assessment sites for monthly supply of ARVs

Communication issues at assessment sites	• Few or no telephones • No fax machines or photocopy machines • No electricity (one clinic) • Shortage of computers or poor connectivity causing back log in data collection • Shortage of data clerks

Space issues	• Lack of sufficient consulting rooms • Lack of space for large drug readiness training classes • Lack of waiting room space for ART patients

Thus, in their comments on the proposed intervention and in order to address some of the problems outlined in Table 2, such as nurse workload and transport difficulties for patients, many of the staff felt that more elements of HIV care, including drug readiness training and monthly collection of ARVs, needed to be integrated into primary care services. Furthermore, these elements of care needed to be available not only within the ART clinic but also in surrounding primary care clinics referring patients to these ART sites. Task shifting of pre-ART care from ART nurses to primary care nurses at ART sites, as initially envisaged in discussions with management, was thus reformulated as a step-wise integration of the following six elements of comprehensive HIV care into all primary care services both within the ART clinics and those at clinics referring patients to the ART nurses at the ART sites: voluntary counselling and testing; initial CD4 count; routine HIV care for patients not yet eligible for ART; drug readiness training for patients initiating ART; baseline blood tests for patients initiating ART; and monthly ART care for stable patients. This 'decentralisation checklist' was included in the implementation toolkit.

A meeting was also held to gather the views of primary care nurses in the 16 ART sites. These nurses were concerned about the burden of HIV disease in their patients, were keen to be involved in the programme, and felt capable of providing comprehensive HIV care. However, they were also concerned about the increased workload this would create for healthcare providers in already overloaded and understaffed primary care services.

### Components of the intervention

The main components of the intervention are discussed below and are summarised in Table 3, where they are compared with standard of care support at control clinics.

**Table 3:** Components of the intervention compared to standard care at control clinics

Intervention component	Intervention clinics (n = 16)	Control clinics (n = 15)
STRETCH Coordinator	• Teaching in the Free State ART training programme alongside ART programme doctors • Available for clinical advice for all staff in ART sites • Initial training and support of nurse trainers at intervention sites • Providing extra support to nurses prescribing ART at intervention sites • Facilitating the establishment of local management teams to implement the intervention	• Teaching in the Free State ART training programme alongside ART programme doctors • Available for clinical advice for all staff in ART sites

Regulatory environment for prescription of ART	• Pharmaceutical and Therapeutics Committee of the Free State Department of Health gave permission for professional nurses at intervention sites to initiate and repeat prescriptions of ART for adults identified as eligible for nurse management.	• Only doctors were allowed to initiate and repeat prescriptions three or six monthly for patients needing ART

Nurse Training	• All professional nurses completed two-week ART training and on-site training in PALSA PLUS guidelines--six to eight sessions in total • 16 PALSA PLUS trainers, one for each clinic, trained in use of STRETCH guidelines (TtTtT) • All professional nurses offered on-site training in the use of STRETCH guidelines to identify patients eligible for nurse management-four sessions in total	• All professional nurses completed two-week ART training and on-site training in PALSA PLUS guidelines-six to eight sessions in total

Patient management guidelines for nurses	• Special 2007 STRETCH Free State edition of PALSA PLUS guidelines with extra STRETCH guidelines for nurse initiation and repeat prescription of ARVs issued to all staff at intervention sites	• Standard 2006 edition of PALSA PLUS issued to all staff at control sites during training in 2006 or 2007

Management support	• STRETCH team established at each intervention site to manage the introduction of changes in clinic function during the intervention • Local area management support teams were set up to support the integration of aspects of comprehensive HIV care into the services of these primary care clinics referring patients to the intervention site	• Standard management support by clinic supervisor, district ART coordinator and local area manager

Implementation guideline	• STRETCH Toolkit issued to STRETCH teams at 16 intervention clinics to assist the teams in implementing the intervention	• None

Phased introduction	• Phase one: Training and establishment of STRETCH teams at each intervention site • Phase two: Nurse repeat prescription of ART for patients on ART for six months or more and eligible for nurse management • Phase three: Nurse initiation of ART for adults eligible for nurse management	• None

### The STRETCH coordinator

A provincial STRETCH coordinator (KU), a family medicine practitioner with experience in the management of HIV/AIDS and tuberculosis, was appointed and had the following responsibilities during the intervention: further developing the intervention in consultation with staff at management and clinic level; involvement in initial training and continuing support of nurse training at intervention sites; teaching in the Free State ART training programme alongside ART programme doctors; helping to provide clinical advice to all ART sites; providing extra support to nurses prescribing ART at the intervention sites; and facilitating the establishment of management teams to oversee the implementation of the intervention. The involvement of the STRETCH coordinator in teaching in the ART programme and helping to provide clinical advice to all ART sites was not initially envisaged as part of the intervention, but was included at the request of the province because of the shortage of doctors available to provide this support.

### Regulatory changes

Although there was no official national policy prior to the trial on nurse prescription of ART, two pieces of national legislation supported such prescription [40,41]. The Free State Pharmaceutical and Therapeutics Committee gave permission for professional nurses in the province to initiate and repeat ART prescriptions for adults during the trial. This permission was conditional on these nurses completing appropriate training and working at one of the 16 intervention clinics. Usual care continued at the 15 control clinics where only doctors were allowed to prescribe ART.

### Nurse training

Table 4 summarises the characteristics of the ART training available to nurses in all clinics across the province and the training offered as part of the intervention. The details of these training programmes are described below.

**Table 4:** Characteristics of various nurse trainings available as standard of care in all ART and primary care sites compared with training offered at intervention clinics during STRETCH intervention

	Free State Department of Health ART course (Standard training)	PALSA PLUS training (Standard training)	STRETCH Training (Additional training in intervention clinics)
Description	Two- week training course comprising one week of lectures and one week of practical training	One- to two-hour sessions weekly or fortnightly of case scenario-based interactive training in use of PALSA PLUS guidelines (six to eight sessions in total)	One- to two-hour sessions weekly or fortnightly of case scenario-based interactive training in use of PALSA PLUS STRETCH guidelines (four sessions in total)

Trainers	Senior doctors, pharmacists dieticians and social workers working in ART programme	Middle level nurse managers trained as PALSA PLUS trainers	Middle level nurse managers trained as PALSA PLUS and STRETCH trainers

Trainees	Doctors, professional nurses enrolled nurses pharmacists and social workers involved in providing primary care services at hospitals and clinics across the province	Professional and enrolled nurses and ancillary staff at all intervention and control clinics and primary care clinics throughout the province.	All professional nurses (whether appointed to ART or primary care posts) at 16 intervention sites only

Setting	Local classrooms located throughout the province to which lectures are broadcast. Local ART sites during practical training	Training sessions held at the clinic	Training sessions held at the clinic

Mode of delivery	Lectures broadcast live from central studio with limited telephone interaction. Face-to-face with staff at ART sites during practical training	Face-to-face small group facilitative work	Face-to-face small group facilitative work

Intensity and duration	Full day training for one week of lectures and one week of practical training	One to two hours once every week or two weeks for two to three months	One to two hours once every week for four weeks

### Standard of care training in all clinics

Since 2005, the Free State Department of Health has been running a regular two-week ART training course for staff in ART and other primary care clinics. This course combines one week of lectures broadcast to classrooms throughout the province and a one-week placement at an existing ART site. Regular maintenance training is also conducted in the districts and in weekly lectures broadcast to staff in these classrooms. Clinical support was available to staff at all ART sites from doctors at treatment sites, specialists at a tertiary level AIDS clinic and the STRETCH coordinator.

At the time of the trial, PALSA PLUS training was being rolled out to all provincial primary care clinics, including all ART assessment sites [27]. This model of training involves equipping nurse managers to conduct outreach training for nurses at clinics in their area. Nurse managers are trained in a one week course known as Training the Trainer to Train (TtTtT) [25]. Adult education models are used to fully integrate experiential learning on how to facilitate small group training using case scenarios, while enabling the trainers to become familiar with the contents of the guideline. These nurse managers in turn conduct outreach training onsite, in short sessions over several weeks, using these case scenarios to facilitate nurses engaging with the PALSA PLUS guideline. This training has been shown to be effective in improving quality of care and minimises disruption to clinic services [26,27]. Thirty of the 31 ART sites in the STRETCH trial had completed PALSA PLUS training before the trial began and plans were made to train staff at the outstanding clinic.

### Training at intervention clinics

The PALSA PLUS model of training was expanded to include extra training in nurse prescription of ART. One established PALSA PLUS trainer was identified for each of the 16 intervention clinics. All had been trained in ART, and three had experience working in ART sites. These trainers were either clinic supervisors or local programme coordinators regularly visiting these clinics in a supervisory capacity. They participated in a two and one-half-day training on: how to train nurses in the ART protocols contained in the STRETCH edition of the guidelines by using four case scenarios; and the staff role changes needed as part of the intervention, as described in the toolkit. We anticipated that nurse confidence might be severely compromised if patients who were started on ART by nurses developed severe side effects. The case scenarios were therefore also used to impart basic skills for trainers to debrief nurses. The training was led by three facilitators from the research team: two nurses experienced in adult and nurse education who had been involved in developing the PALSA PLUS training (GF and PM), and the STRETCH coordinator.

The trainers then trained all nurses at the 16 intervention clinics, including designated ART nurses and those working in primary care, commencing in August 2007. A minimum of four educational outreach trainings, one of which was supported by the STRETCH coordinator, were conducted at each clinic, and most of these sessions were completed by October 2007. The trainers continued to support the nurses and train those who were newly appointed or had not attended all the initial sessions, but the regularity of these visits varied and depended on their other supervisory responsibilities.

All doctors supporting the intervention sites were oriented by the STRETCH coordinator using the guidelines and case scenarios. Doctors working in the five combined sites were able to provide clinical support to the nurses. However, at the other eleven assessment sites, where doctors only worked at distant treatment sites, they were less able to provide support. Additional clinical support was also provided by the STRETCH coordinator via telephone or during clinic visits. These visits took place typically once every four months in the first twelve months of the trial and less frequently after that.

### Patient management guidelines for nurses

Nurses working in all primary care clinics including all ART sites had access to and were receiving training in the use of the PALSA PLUS guidelines (see above). A STRETCH edition of the PALSA PLUS guideline, containing algorithms for nurse initiation and management of adults on ART, was distributed to all nurses in the 16 intervention clinics and used in outreach training by the STRETCH trainers. The algorithms were developed in consultation with clinicians in the province and with reference to the Integrated Management of Adolescent and Adult Illnesses guideline [42]. Thus, adults with a CD4 <50, Stage 4 HIV, previous ARV treatment, who were on tuberculosis (TB) or other chronic medication, were bedbound, or who were pregnant were identified as potentially complicated cases that needed to be initiated onto ART by a doctor. All other adults eligible for ART could be initiated by nurses. Similarly, a decreasing CD4 count, detectable viral load, or clinical problems in a patient already receiving ART were criteria for doctor management, while all other patients could be managed by a nurse. (The ART algorithms are included in Additional file 1)

### Phased introduction

The intervention was implemented in phases to support logistical changes such as the dispensing of nurse ART prescriptions and to allow nurses to build confidence and skills in ART prescriptions. The three phases of implementing the intervention were: the training of nurses in ART prescription and setting up of management support teams; nurse re-prescription of ART for stable patients; and nurse initiation of ART for uncomplicated new patients. The timing of progress through the stages was determined by staff in the STRETCH teams at each individual clinic.

### Implementation guideline

Because of the complexity of the intervention, the research team developed an implementation guideline called the STRETCH Toolkit and distributed copies to all intervention sites. The Toolkit contained the decentralisation checklist (as outlined above), descriptions of the different phases of the study, as well as details about the changing roles of all staff members in each phase and useful advice on communicating these changes to the community. It also contained important documents and information, such as contact numbers for doctors and nurse managers of all the clinics in the trial and relevant managers in the provincial department, along with copies of documents authorising nurse prescription of ART. (The STRETCH Toolkit is included in Additional file 2)

### Management support

Standard support was provided to all ART sites by two to three monthly visits from district ART coordinators (who had district wide responsibility for the ART programme) and monthly visits from clinic supervisors (who were responsible for overall primary care services in a local group of clinics). Meetings between clinic managers (in charge of each clinic) and local area managers (who had overall responsibility for health services in that local area) are typically held at one- or two-month intervals.

During phase one of the intervention, STRETCH teams were convened by the STRETCH coordinator at each of the intervention clinics. These teams usually comprised the clinic manager, one clinic nurse representing ART services and one representing primary care, and the pharmacist or pharmacy assistant, as well as staff from the treatment site and the district ART coordinator. These teams were given copies of the STRETCH Toolkit and were tasked with implementing changes at the clinic during the intervention. One of these tasks, as outlined in the decentralisation checklist, was to assess the state of integration of comprehensive HIV care into primary care services, and which further elements of HIV care needed to be integrated into these services (Table 1).

Thirteen of the intervention clinics had patients referred for ART from other primary care clinics in their area. In four of these intervention clinics, local management had already started implementing the integration of all six elements of HIV care into the primary care clinics. In the other nine intervention clinics, the STRETCH team identified the need to integrate further elements of HIV care into these referring clinics. Local area management teams were then convened for seven of the nine clinics. In the remaining two clinics management support was difficult to mobilise. These teams usually comprised the local area manager, the manager of the intervention site, facility managers of all referring primary care clinics, and the local ART pharmacist. They were able to evaluate capacity to integrate further elements of HIV care into the referring clinics by assessing staffing and training needs, space for drug readiness training classes, and ability to store and transport ARVs--all of which were the type of practical issues identified by staff (Table 2). The STRETCH coordinator's responsibility was to convene these management teams and assist at the first one or two meetings. It was then the team's responsibility to decide which elements of HIV care could be integrated at which primary care clinics and to implement these decisions.

## Discussion

One of the distinctive features of this intervention was the participation of clinic staff and all levels of management in many stages of its development and implementation. First, the trial was set up at the request of senior management to address the problem of high mortality rates among patients eligible for ART and awaiting access to treatment. In the national environment of ambivalence to nurse ART-prescription that existed at the start of the trial, senior management support was crucial to developing and implementing the intervention. Second, senior management, middle management, and clinic staff were involved in an iterative process of assessing the barriers facing patients and staff with regard to accessing ART, and then tailoring the intervention to be relevant and implementable. Management concerns about the complexity of the intervention led to the development of an 'Implementation Toolkit.' The types of problems outlined by staff (Table 2) and their insight into possible solutions led to the reformulation of integration in the context of ART rollout as the flexible, progressive integration of pre-ART and ART care into all primary care services referring to intervention sites. Third, staff at local area and clinic level were involved in the teams tasked with implementing the intervention, with support from the STRETCH coordinator. STRETCH teams were tasked with assessing readiness for different phases of the intervention and with implementing the changes at clinic level. Local management teams assessed capacity and arranged for primary care services to take on aspects of pre-ART and ART care.

The strong participation of clinic staff and managers in intervention development and implementation could be seen as an example of how features of participatory action research can be integrated into trial intervention design and implementation. It has been suggested that this approach to intervention design may make complex health interventions both more effective and more easily reproducible in other settings [43]. This is congruent with evidence from a systematic review that suggests that interventions tailored to prospectively identified barriers have a greater likelihood of improving professional practice than interventions with no such tailoring [44]. However the review also notes that further work is needed on methods to identify barriers and tailor interventions to address them. The participatory approach used here is also in line with calls to involve the district health systems in efforts to deliver comprehensive HIV care [8,17,45]

One of the weaknesses of the development of this intervention is that, while staff at the ART sites were involved in initial discussions, staff at the primary care clinics referring patients to these sites were not. However, as part of the implementation, managers of these primary care clinics were included as members of local management teams and were then able to give their input, assess capacity issues, and make workable plans for the integration of HIV care into their clinic services.

A second change technique used to facilitate uptake of the intervention was educational outreach. This approach was the basis for the training of professional nurses in the intervention clinics. The PALSA PLUS training model, on which the STRETCH intervention was based, draws on adult education principles and the outreach education approach, and has been shown to be effective in changing nurse clinical practice in study setting and more widely [26,27,46]. The trainers chosen to implement this training were local staff members--another facet of active participation in the implementation. Many of the 16 STRETCH trainers were themselves clinic supervisors and had also been PALSA PLUS trainers. As part of this trial, they trained the professional nurses at the clinics for which they provided supervision.

The STRETCH coordinator also functioned as an 'agent of change' in this intervention, playing a role in facilitating the active participation of staff in, firstly, the process of developing and reformulating the intervention so that it was implementable and responsive to local conditions in the clinics and, secondly, in establishing local teams to implement the intervention actively. The coordinator was appointed by the research team but based in the provincial health department. This allowed her to facilitate communication between the research team and provincial staff and act as a 'problem solver.' The coordinator was also able to provide ongoing support to nurses, doctors, and trainers because of her previous clinical experience. All of these roles have been acknowledged as important functions of external facilitation in the implementation of complex health interventions [47]. Models of implementation also acknowledge the overlap between outreach educators, which formed one component of this intervention, and facilitation, which formed another component. These models suggest that facilitators take on a wider range of roles than outreach educators, including the use of a greater range of enabling approaches to help support practice change and mediate between stakeholders [48].

## Conclusion

This paper describes the development and content of the STRETCH intervention intended to improve access to ART. This complex intervention incorporates three processes: participatory action research, educational outreach, and external facilitation to change the practice of nurses in primary care settings in South Africa. The effects of the intervention are now being evaluated in a pragmatic randomised controlled trial. To evaluate the degree to which the intervention was implemented as intended [43,49], a qualitative process evaluation of the trial was conducted. In addition, the integration of HIV care into primary care services was monitored using a semi-quantitative questionnaire. The findings of these parallel studies will contribute to understanding the effects of the intervention described in this paper.

## Competing interests

The authors declare that they have no competing interests.

## Authors' contributions

LF, SL, MB, MZ, CL, and EB were involved with initial conception, design and development of the trial and reviewing the manuscript. LF, KU, GF, and PM were involved in developing and implementing the intervention and writing the manuscript. DvR and WM were involved with writing and reviewing the manuscript. CC and DG reviewed the manuscript. All authors read and approved the final manuscript.

## Supplementary Material

Additional file 1 **ART algorithms**. Algorithms for initiation and management of patients on antiretroviral therapy included in the STRETCH edition of the PALSA PLUS guideline that was used in intervention clinics during the STRETCH trial. Click here for file

Additional file 2 **STRETCH Toolkit**. STRETCH Implementation toolkit developed by the research team to assist clinic staff in implementing the STRETCH intervention. Click here for file
