# Supplementary material for: Task shifting and integration of HIV care into primary care in South Africa: The development and content of the streamlining tasks and roles to expand treatment and care for HIV (STRETCH) intervention
Source: Implement Sci. 2011 Aug 2;6:86. doi: 10.1186/1748-5908-6-86 (PMC3163620; doi:10.1186/1748-5908-6-86)
Supplement: Additional file 1 — ART algorithms. Algorithms for initiation and management of patients on antiretroviral therapy included in the STRETCH edition of the PALSA PLUS guideline that was used in intervention clinics during the STRETCH trial. [file 1748-5908-6-86-S1.PDF]

ENROLMENT IN THE ARV PROGRAMME

- ARVs prevent and treat AIDS. ARVs are for life.
- **Clients need ARVs if CD4 ≤ 200** or **stage 4 HIV. If pregnant, client needs ARVs if CD4 ≤ 350** or **stage 4 HIV.**
- All clients need routine HIV care (see page 17)
- At the first visit: - Assess eligibility for nurse-managed or doctor-managed ARVs (step 1)
  - Prepare all clients for ARVs with steps 2–6

Step 1. Assess eligibility for nurse-managed or doctor-managed ARVs

- CD4 51-200 and
- Stage 1, 2 or 3 HIV and
- ARV-naïve (no previous ARVs or ARVs ≤1 month) and
- Able to walk unaided and
- Only using co-trimoxazole ± multivitamins and
- Not pregnant and
- Weight > 40kg and BMI< 28

For nurse-managed ARVs

- CD4 ≤ 50 or
- Stage 4 HIV or
- Previous use of ARVs (>1 month) or
- Bed- or wheelchair-bound or
- Using medication other than co-trimoxazole and multivitamins or
- Pregnant (refer same week for Drug Readiness Training) or
- Weight < 40kg or BMI > 28

Refer for doctor-managed-ARVs (same week if CD4 ≤ 50 or pregnant)

Step 2. Exclude TB. Always look for TB symptoms.

Exclude TB
Go to page 6

Investigate for TB if any of the following are present:

- Cough • 2 weeks or
- Weight loss • 1.5kg in 4 weeks or
- Drenching night sweats or fever • 2 weeks or
- Chest pain or
- Blood stained sputum

} Send sputa for two smears (see pages 6 - 8 for diagnosing TB)

If symptomatic, do not commence ARVs until TB has been excluded. If unsure, refer to doctor.

Step 3. Assess clinically

Routine Care
Weight
Exclude TB
Review CD4
Update Stage
STI screen
Condoms
Pap

- ARVs are not an emergency treatment: HIV emergencies are usually due to opportunistic infections.
- Assess for opportunistic infections or other HIV-related diseases.
  - Refer the client with CD4 ≤ 50 or Kaposi's sarcoma same week to doctor for ARVs.
  - Look for and treat **acute severe illness** – stabilize the client before starting ARVs. (See pages 24-35)
  - Ask about
    - **peripheral neuropathy** (pain, burning/ 'heat' or tingling in the hands or feet). (See page 31)
    - **depression.** (See page 35)
    - **pregnancy** (refer to doctor for ARVs same week)
- Assess nutritional status, calculate BMI (see page 18)

Step 4. Discuss contraception and safe sex

- Discuss your client's plans for a family. If required, advise reliable contraception (injectable contraceptive plus condoms).
- **Efavirenz causes birth defects. Women of child-bearing age must receive nevirapine instead.**
- Unsafe sex on ARVs can still transmit HIV and carries the risk of reinfection with different strains of HIV. This can lead to treatment failure.
- Encourage the use of condoms. Encourage your client to have only one partner.

Step 5. Assess blood results

- All clients need a baseline ALT.
- Normal range < 40 IU/ml. If result not within normal range, refer to doctor for assessment and to start ARVs

Step 6. Start Drug Readiness training at the same time as clinical work-up

One session per week for three weeks – clients must complete all three sessions before starting ARVs
If client is pregnant, she should complete drug readiness training within one to two weeks

- Session One: Disclosure and Positive Living
- Session Two: Basics of HIV, CD4 and viral load; Co-trimoxazole prophylaxis
- Session Three: Opportunistic Infections, ARV Treatment Plan, Adherence

Encourage attendance by treatment 'buddy' (friend or family member)

Step 7. Assess readiness to start treatment

ARVs are not an emergency treatment. Clients must be clinically stable, psychologically prepared and adherent before starting treatment.

Clinically ready?
Able to walk unaided
No TB symptoms
No acute illness
Normal baseline ALT

Adherent?
Takes co-trimoxazole/ multivitamins as instructed
Attends appointments reliably
Understands the importance of adherence
Plans for regular attendance and adherence

Socially ready?
Treatment buddy
Support group recommended
No alcohol abuse
Contraceptive/ condoms

If yes to all **the above, client is ready to start nurse-managed ARVs. If no to** any **of the above, refer for doctor-managed ARVs.**

Step 8. Start ARVs – Regimen 1

- The client must always receive 3 different ARVs. Prescribe 3TC and d4T and either:
  - nevirapine for all women of child-bearing age or
  - efavirenz for all men and women not of child-bearing age
- Counsel client about how to take ARVs
- Remind about possible side effects (see page 22)
- Draw baseline viral load
- Continue co-trimoxazole ± multivitamins.
- Schedule clinic follow-up after two weeks

Antiretroviral	Weight	Dose	Frequency
Lamivudine (3TC)	>40kg	150mg	12-hourly
Stavudine (d4T)	40-60kg	30mg	12-hourly
Nevirapine	>40kg	200mg	once daily for 2 weeks, then 12-hourly[1]
Efavirenz	>40kg	600mg	24-hourly - the same time every night

ces liver enzymes responsible for its own metabolism. Step-wise introduction helps to avoid sub-therapeutic levels and reduce the risk of skin rash and hepatitis.

MONITORING THE CLIENT ON ARVs

Routine Care
Weight
Exclude TB
Review CD4
Update Stage
STI screen
Condoms
Pap

Follow-up appointments for client on ARVs. ARVs are for life.

- Check for adherence and reliable attendance. Re-issue medication (ARVs, co-trimoxazole) monthly.
- Check safety bloods according to schedule below
- Monitor response to ARVs (see below)
- Look for ARV side-effects and review safety bloods results. (See page 22)

Week	2	4	8	12	24 (6 months)	48 (12 months)	6-monthly thereafter	12-monthly thereafter
Safety bloods per ARV	• ALT (NVP)<br>• Increase **NVP to 12-hourly if client well**	• ALT (NVP)<br>• FBC + diff (AZT)	• ALT (NVP)<br>• FBC + diff (AZT)	• FBC + diff (AZT)	• ALT (NVP)<br>• FBC + diff (AZT)<br>• Fasting cholesterol and triglycerides (LPV/r)	• ALT (NVP)<br>• FBC + diff (AZT)<br>• Fasting Glucose (LPV/r)	• ALT (NVP)<br>• FBC + diff (AZT)	• Fasting cholesterol & triglycerides (LPV/r)<br>• Fasting glucose (LPV/r)

Monitoring response to ARVs

Response to ARVs is assessed clinically, virologically (with viral load, VL) and immunologically (with CD4)

Exclude TB
Go to page 6

Clinical

- After starting ARVs, opportunistic infections can occur, especially if CD4 < 100. Look for signs of infection, particularly TB, at each visit.
- Weight: - Investigate > 1.5kg weight loss.
  - Weight gain > 10kg or to BMI > 28 increases risk of lactic acidosis. Refer to doctor.

CD4

- Check 6-monthly.
- Response varies from client to client.
- Some CD4s may never rise.
- Refer for doctor review if CD4 falls and new opportunistic infections or VL > 400.
- If well and CD4 > 200 stop co-trimoxazole

Viral load

- Check 6-monthly.
- Should be < 400 or 'lower than detectable limits'.
- If > 400 refer to adherence counselor same day and refer to doctor for next ARV appointment.

Treatment failure

- Refers to a VL persistently > 400 with or without the occurrence of opportunistic infections.
- The client will need to change to a new ARV regimen, usually regimen 2.
- The most common cause of treatment failure is low adherence.

Regimen 2 ARVs	Weight	Dose	Frequency
Zidovudine (AZT)		300 mg	12-hourly
Didanosine (ddI)[1]	< 60 kg<br>> 60 kg	250 mg<br>400 mg	Once a day
Lopinavir/ ritonavir (LPV/r)[2]		400/100 mg	12-hourly

Approach to low adherence (See page 44 for an approach to adherence counseling)

More than 95% of ARV doses must be taken to avoid development of resistance.

- Educate on the importance of adherence and dangers of resistance
- Re-explain treatment schedule (12-hourly doses including weekends)
- Consider adherence aids (pillboxes, diaries)
- Ask about drug-related side-effects or alcohol abuse
- Refer client to adherence counselor
- Insist on participation in a support group
- See the client more frequently (weekly instead of monthly)
- Arrange a home visit

Inform ARV site doctor and nurse of low adherence so that CD4 and VL can be interpreted accordingly.

[1] Dissolve at least 2 tablets in water. Take on an empty stomach 1 hour before food or medication.
[2] Take with food to improve absorption. Store in a cool, dry place. (< 25 °C)
