# Supplementary material for: Task shifting and integration of HIV care into primary care in South Africa: The development and content of the streamlining tasks and roles to expand treatment and care for HIV (STRETCH) intervention
Source: Implement Sci. 2011 Aug 2;6:86. doi: 10.1186/1748-5908-6-86 (PMC3163620; doi:10.1186/1748-5908-6-86)
Supplement: Additional file 2 — STRETCH Toolkit. STRETCH Implementation toolkit developed by the research team to assist clinic staff in implementing the STRETCH intervention. [file 1748-5908-6-86-S2.PDF]

STRETCH

Streamlining Tasks and Roles to Expand Treatment and Care for HIV

Implementation Toolkit 2007

Department of Health
Departement van Gesondheid
Lefapha La BopheloBo Botle

FOREWORD

The STRETCH intervention is an important pilot intervention in ARV clinics in the Free State and is aimed at increasing access to treatment for the many HIV-infected patients in our province who need antiretroviral therapy.

We recognise the tremendous work done so far by all our healthcare workers in rolling out ARVs across the province, but we also see that we need to get many more patients onto ARVs if we are going to make an impact on the toll HIV is taking in our communities.

To this end the STRETCH programme is one way of steamlining and increasing provision of HIV care by redefining the roles of different health care workers and reintegrating HIV care back into the primary healthcare system.

This is being done as a research pilot programme in partnership with the UCT Lung Institute, so that we can monitor the effect of this new programme to see if we can expedite access to ARVs for people who need them, without compromising standards of care.

I recommend this programme to you and support its implementation and look forward to seeing the results.

MS SRO KHOKHO
Acting Executive Manager
Strategic Health Programmes & Medical Support

Department of Health
Departement van Gesondheid
Lefapha La BopheloBo Botle

CONTENTS

**What is STRETCH?**	2
**STRETCH Overview**	3
Prescribing/Dispensing and Issuing of ARVs during STRETCH	5
**The STRETCH Support Team**	6
Phase 1	7
Phase 2	8
Phase 3	9
Maintenance and Support	10
Tips on Streamlining Care	11
Tips on Managing Staff Shortages	12
**Tasks and Roles during STRETCH**	13
PHC Services Nurse	13
ARV Nurse	14
ARV Doctor	15
Pharmacist/Pharmacy Assistant	16
Admin Clerk	17
Data Capturer	18
PALSA PLUS trainer	19
**STRETCH Resources**	21
Suggested Timeline of Activities	20
Decentralisation Checklist	21
Equipment and Supplies Checklist	22
Suggested Programme for Orientation Workshop	23
**STRETCH Documentation**	
Provincial Permission for STRETCH	24
Ethics Permission for STRETCH Trial Evaluation	25
Pharmaceutical and Therapeutics Committee Permission for Expanded Prescribing Provisions	26
**Contact lists**	27

WHAT IS STRETCH?

STRETCH is a multifaceted health systems intervention comprising:

- Algorithms to triage HIV patients eligible for ARVs for nurse- or doctor-managed care (included in a special edition PALSA PLUS guideline).
- PALSA PLUS educational outreach training in the new guideline.
- Expanded prescribing provisions to permit trained nurse practitioners[1] to prescribe ARVs.
- Re-defining roles of clinical staff
  - Primary healthcare services: pre-ARV HIV care.
  - ARV nurses: monitoring of stable ARV patients, ARV initiation in selected adults.
  - ARV doctors: manage complex cases and review problem cases.
- This system's toolkit: a handbook for managers on how to implement STRETCH.
- Provincial STRETCH co-ordinator.
- STRETCH facility support teams – to facilitate changes and provide support.
- Community awareness by community health workers.

STRETCH is ***not*** just nurse-initiated ART, nurses doing doctors' work, excluding doctors or a quick fix.

STRETCH aims to:

- Provide high quality HIV and ARV care while expanding ARV treatment access.
- Decentralise care and integrate HIV care into primary care.
- Consolidate care for most clients to the clinic/assessment site to reduce traveling between facilities and to avoid fragmented care.
- Enable doctors to see complex cases.
- Provide a sustainable model of care and support health workers working together to avoid burn-out.

STRETCH will be introduced in 3 phases:

1. Site preparation including decentralisation of HIV care according to provincial policy (e.g. rollout of CD4 staging).
2. Consolidation of decentralisation of HIV care to PHC services and ARV monitoring to ARV nurses.
3. Initiation of ARV treatment by nurses in selected cases.

[1] Only selected nurse practitioners at STRETCH facilities may prescribe ARVs. See page 5 for details.

HOW TO USE THIS TOOLKIT

- As a **handbook**! The organisation of care in a clinic depends on many factors including its size, location, distance from referral hospital and staffing. The recommendations in this toolkit ***must*** be tailored for each STRETCH clinic. Some clinics will have more changes to make than others to achieve integrated decentralised HIV care.

- To **inform others** including managers and clinic staff. The overview of the programme (pages 3-4) provides a useful summary, and descriptions of everyone's tasks and roles during each phase may also help provide clarity.

- As a **resource**. The toolkit includes useful checklists (pages 21-22), contact numbers (pages 27-29) and documents (pages 24-26) which you may need during your interactions with other mangers and clinical staff.

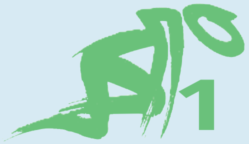

Phase 1: Site preparation (3 months)

KEY ACTIVITIES
- PALSA PLUS training to all clinic nurses to ensure all equipped to manage HIV and TB.
- Convene support team for each STRETCH facility to initiate system changes for Phases 2 and 3.
- Start decentralisation of routine HIV care according to provincial policy (e.g. VCT and CD4s at local clinic).

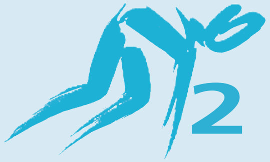

Phase 2: Decentralisation of HIV care to PHC services nurses and ARV monitoring to ARV nurses (2 to 3 months)

KEY ACTIVITIES
- Consolidate efforts to decentralise routine HIV care to PHC services nurses.
- ARV monitoring decentralised to ARV nurses at STRETCH facilities.
- STRETCH support team to meet regularly (weekly for 3 weeks, thereafter 2 weekly) & PALSA PLUS training to continue.

Initial assessment by PHC services nurse with ***same day*** CD4 draw

CD4 result appointment with PHC services nurse

CD4 $\leq$ 200 and/or AIDS and not pregnant ***or***
Pregnant and CD4 $\leq$ 350 and/or AIDS

Refer to ARV nurse for ARVs. Fast-track if pregnant

Refer to doctor at treatment site for ARVs
Continue work-up for ARVs:
CD4 if not yet done, TB screen, RPR, Preg test, ALT, Pap etc.

Monitoring on ARVs
ARV nurse reissues/represcribes ARVs and other meds, draws **and** interprets all bloods, supports adherence, refers selected cases to doctor.
ARV doctor reviews cases referred by ARV nurse and, if appropriate, changes drugs.

CD4 $\geq$ 200 and no AIDS and not pregnant ***or***
Pregnant and CD4 > 350 and no AIDS

6 monthly review by PHC services nurse
Referral to PMTCT programme

Routine HIV care can prevent AIDS
PHC services nurse to monitor CD4
CD4 251 to 499: recheck 6 monthly
CD4 $\geq$ 500: recheck 12 monthly

Phase 3: Initiation of ARV treatment by nurses in selected cases

KEY ACTIVITIES

- ARV nurses triage all clients referred for ARVs & initiate treatment in selected cases. Others are referred to doctors at the treatment site as before.
- STRETCH support team to meet weekly for first 6 weeks, thereafter 2 weekly & PALSA PLUS training to continue.

Initial assessment by PHC services nurse with same day CD4 draw

CD4 result appointment with PHC services nurse

CD4 $\leq$ 200 and/or AIDS and not pregnant ***or***
Pregnant and CD4 $\leq$ 350 and/or AIDS

Refer to ARV nurse for ARVs. Fast-track if pregnant.

Triage clients according to PALSA PLUS guideline (STRETCH edition)
and work-up for ARVs
For nurse or doctor-managed ARVs?

For nurse-managed ARVs

Initiate ARVs and monitor according to guideline

For doctor-managed ARVs

Refer to doctor at treatment site for ARVs

Monitoring on ARVs
ARV nurse reissues/represcribes ARVs and other meds, draws and interprets all bloods, supports adherence, refers selected cases to doctor.
ARV doctor reviews cases referred by ARV nurse and, if appropriate, changes drugs.

CD4 $\geq$ 200 and no AIDS and not pregnant ***or***
Pregnant and CD4 > 350 and no AIDS

6 monthly review by PHC services nurse
Referral to PMTCT programme

Routine HIV care can prevent AIDS
PHC services nurse to monitor CD4:
CD4 251 to 499: recheck 6 monthly
CD4 $\geq$ 500: recheck 12 monthly

TIMELINES

- It is recommended that STRETCH be introduced over a period of 4 to 6 months in a clinic. This will depend on the number of changes that need to be made in a clinic to achieve integrated decentralised HIV care.
- Phase 1 has already started in all STRETCH clinics, but will need to be consolidated before proceeding to Phase 2.
- The duration of Phase 2 depends on the extent of verticalisation in the clinic, and the confidence of ARV nurses in monitoring clients on ARVs. A period of 2 to 3 months is recommended.
- Schedule activities (PALSA PLUS training, STRETCH support team meetings, start dates for phases 2 and 3, orientation workshops etc.) to ensure implementation proceeds smoothly. See page 20 for a suggested timeline.

PRESCRIBING/DISPENSING AND ISSUING OF ARVS DURING STRETCH

- Only nurses working in selected STRETCH clinics may be considered for prescribing ARVs until further notice.
- In order to prescribe ARVs nurses must fulfill **all** of the following criteria:
  - Professional/Senior Professional/Chief Professional nurse authorised to prescribe medication at the clinic.
  - Completed provincial ARV training.
  - Completed or receiving outreach training in the PALSA PLUS guideline (STRETCH edition).
- All nurses who fulfill these criteria must be registered with the STRETCH provincial co-ordinator in order to be able to prescribe ARVs. Fax certificates for above training to Dr Kerry Uebel at 051 4081961.
- These nurses may only prescribe ARVs according the clinical criteria in the PALSA PLUS guideline (STRETCH edition).
- Nurses may not dispense ARVs. Scripts should be faxed to pharmacists/pharmacy assistants at the treatment site or central pharmacy for dispensing. Once dispensed they will be sent to the clinic for issuing.
- Permission for nurses to prescribe ARVs under these conditions has been provided by the Free State Pharmaceutical and Therapeutics Committee (see page 26).

	**Initiate**	**Renew same prescription**	**Change prescription**	**Dispense**	**Issue**
Definition	*Complete first prescription for ARVs*	*Renew same ARVs at same doses for a further period (usually 3 months)*	*Change regimen or dose of ARVs*	*Attach client's personal details to packets of medication*	*Supply client with dispensed medication*
**Phase 1**	Doctor	Doctor	Doctor	Pharmacist<br>Pharmacy Assistant	Pharmacist<br>Pharmacy Assistant<br>ARV or PHC services nurse
**Phase 2**	Doctor	Doctor<br>ARV nurse (selected cases)	Doctor	Pharmacist<br>Pharmacy Assistant	Pharmacist<br>Pharmacy Assistant<br>ARV or PHC services nurse
**Phase 3**	Doctor<br>ARV nurse (selected cases)	Doctor<br>ARV nurse (selected cases)	Doctor	Pharmacist<br>Pharmacy Assistant	Pharmacist<br>Pharmacy Assistant<br>ARV or PHC services nurse

THE STRETCH SUPPORT TEAM

- The purpose of the STRETCH Support Team is to ensure that the multiple components of STRETCH (PALSA PLUS training, reversal of vertical HIV care, down-referral of stable ARV clients, initiation of ARVs in selected cases) are actually implemented at the STRETCH facility.
- Communication is key to successful implementation and requires that the STRETCH Support Team meet regularly.
- Each STRETCH facility requires its own team.

GETTING STARTED

Who should be in the team?
A provincial STRETCH co-ordinator will convene a group of stakeholders for each STRETCH facility comprising the following:

STAKEHOLDER	ROLE IN THE STRETCH TEAM?
• Facility manager	To oversee changes in client flow in facility and assist with logistical requirements.
• PHC services nurse representative	To re-assume responsibility for routine HIV care, as well as serial CD4 staging.
• ARV services nurse representative	To assume full responsibility for ARV monitoring and initiate ARVs in selected cases.
• ARV doctor	To support ARV nurses.
• ARV co-ordinator	To ensure logistical requirements are met, and report back to district ARV Task Team.
• PALSA PLUS trainer	To train all clinic nurses and support them as their clinical responsibility increases.
• Clinic pharmacist/pharmacy assistant	To ensure adequate supplies of essential drugs (ARVs, cotrimoxazole).
• ARV administrative clerk	To revise filing of records and schedule follow-ups with the appropriate health professional.
• ARV data capturer	To ensure that all relevant records from PHC and ARV nurses are captured.
• Community health worker	To facilitate community buy-in and co-operation.

Who should lead the team?
- If possible, the facility manager should lead the team.
- Effective leadership qualities include being passionate about improving HIV care and ARV access, communication skills, sound decision-making and problem-solving abilities, purposeful planning and effective time management.

- *Teams which get things done meet regularly.*
  - Weekly meetings will be required initially to ensure all the site preparation tasks are completed in Phase 1.
  - More frequent meetings may be required during critical periods (e.g. start of phases 2 and 3).
  - The team will need to downscale its support during the maintenance phase to ensure sustainability in the long-term.
- STRETCH is about consolidating care to the primary care facility. Since most team members come from the facility, it makes sense to hold meetings at the facility itself.

Phase 1: Site preparation and start decentralisation of HIV care

GOAL	ACTIVITIES
To *upskill all nurses* to provide quality HIV and TB care	**PALSA PLUS training**<br>• All clinic nurses should be encouraged to attend on-site training sessions.<br>• Key knowledge includes routine HIV care, interpretation of ARV monitoring bloods and work-up of clients for ARVs including initiation in selected cases.
To ensure *buy-in* of clinic staff, local managers and doctors	**Engage local managers**<br>• Make contact with local managers and doctors through ARV co-ordinator/ District ARV Task Team (e.g. invite local managers/ doctors to a STRETCH team meeting).<br><br>**Orientation workshop for the clinic**<br>• Larger clinics may need a more structured process for raising awareness of STRETCH and ensuring buy-in.<br>• See page 23 for suggested programme for a half-day orientation workshop.
To provide *equipment and supplies* necessary to support the decentralisation of HIV care	See **Equipment and Supplies Checklist** on page 22<br>• Ensure all equipment and supplies are in place before proceeding to Phase 2.<br>e.g. fax machine for faxing prescriptions, adequate storage facilities for extra drugs, extra needles and blood tubes.
To *inform the community* about plans to increase access to HIV care	**Community liaison**<br>• This is the responsibility of the clinic's community health workers.<br>• Key messages for the community in this phase include:<br>- The clinic is planning to increase access to HIV care.<br>- More nurses are being equipped to provide routine HIV care.<br>- In future nurses will start ARVs in uncomplicated adult patients at the clinic.<br>- Complex cases will still be referred to doctors for management.
To *assess readiness* for Phase 2	Have all nurses received **PALSA PLUS training?**<br>Are the necessary **equipment and supplies** in place?<br>Assess progress towards **decentralising HIV care** (see page 21 for Decentralisation Checklist).

Phase 2: Decentralisation of HIV care to PHC services nurses and ARV monitoring to ARV nurses

GOAL	ACTIVITIES
To consolidate *decentralisation of HIV care* to PHC services	**Review Decentralisation Checklist** (see page 21)<br>• Assess progress towards targets for your facility.
To change *follow-up bookings for ARV clients*	**Co-ordinate with Treatment Site through District ARV Task Team/ARV co-ordinator**<br>• Book all ARV follow-up clients from STRETCH clinics for follow-up at the clinic instead of at the Treatment Site (unless complications arise).
To *support clinic staff*	**PALSA PLUS training**<br>• See page 19.<br><br>**ARV nurse doctor partnerships**<br>• The ARV nurse and doctor must work closely to ensure that care is provided at the appropriate level and problems referred without delay.<br>• For this reason try to assign one or two doctors at the treatment site to support nurses at a STRETCH facility. The doctor and nurse should work out how complex cases will be seen (weekly at the clinic or at the treatment site, how to arrange urgent referrals etc).<br>• Encourage feedback from the treatment site (e.g. on appropriateness of referrals, complex cases).<br><br>• Facilitate access to **debriefings by psychologists** through Employee Assistance Programme (EAP). Your PALSA PLUS trainer is also equipped to conduct debriefings.
To *inform clients* about changes in the organisation of HIV care	**Community/client liaison**<br>• Community health workers should inform clients in the waiting room that the organisation of HIV care in the clinic is changing. Daily briefings are suggested (after morning song/prayers).<br>• Key messages for clients in this phase include:<br>- The clinic is planning to increase access to HIV care.<br>- More nurses are being equipped to provide routine HIV care.<br>- Clients on ARVs will be followed-up at the clinic unless complications arise.
To *assess readiness* for Phase 3	Is **monitoring ARVs** at the clinic working? Are the ARV nurses feeling confident about monitoring clients on ARVs?<br>Are **drug supplies** regular?<br>Is there good communication with the treatment site **doctor**? Is she/he seeing complex cases?<br>Are the necessary **equipment and supplies** in place?

Phase 3: Initiation of ARV treatment by nurses in selected cases

GOAL	ACTIVITIES
To consolidate ***decentralisation of HIV care*** to PHC services	**Review Decentralisation Checklist** (see page 21)<br>• Assess progress towards targets for your facility.
To ***decentralise initiation of ARVs in selected cases*** to ARV nurses	**Set date and start ARV initiation at the clinic**<br>• Avoid starting on a Monday or Friday.<br>• Ensure necessary equipment (e.g. fax machine for faxing prescriptions) and supplies (e.g. drugs) are in place (see below).<br>• Consider marking the first time clients start ARVs at the clinic with some form of celebration.
To ***support clinic staff***	**PALSA PLUS training**<br>• See page 19.<br><br>**ARV nurse doctor partnerships**<br>• Communication with the treatment site doctor is vital to support the ARV nurse during the first weeks of phase 3. Ensure that the nurse has the right phone numbers and sufficient phone access, including cellphone access, to contact the doctor when necessary.<br>• Prime the ARV doctor to receive frequent calls from the ARV nurse during the first weeks when clients are expected to present with side-effects.<br><br>• Facilitate access to **debriefings by psychologists** through Employee Assistance Programme (EAP). Your PALSA PLUS trainer is also equipped to conduct debriefings.
To ensure that the necessary ***equipment and supplies*** are in place.	See **Equipment and Supplies Checklist** on page 22.
To ***inform clients*** about changes in the organisation of HIV care	**Community/client liaison**<br>• Community health workers should inform clients in the waiting room about nurses starting to initiate ARVs in selected cases. Daily briefings are suggested (after morning song/prayers).<br>• Key messages for clients in this phase include:<br>- The clinic is planning to increase access to HIV care.<br>- Nurses may commence ARVs in selected adult clients.<br>- Clients on ARVs will be followed-up at the clinic unless complications arise.

MAINTENANCE AND SUPPORT

GOAL	ACTIVITIES
To maintain *decentralisation of HIV care* to PHC services	**Review decentralisation monthly** (see Decentralisation Checklist on page 21)<br>• Are all nurses providing routine HIV care?<br>• Is ARV monitoring and initiation in selected cases being done by the ARV nurse?
To *support clinic staff*	**PALSA PLUS Training**<br>• Follow-up support from PALSA PLUS trainers. PALSA PLUS trainers should revisit STRETCH facilities 4 to 6 weekly during the maintenance phase. See page 19.<br><br>**ARV nurse doctor partnerships**<br>• Maintain open communication channels and promote regular feedback (from ARV task team meetings, on specific clients).<br><br>Regular **clinic/district meetings** to share the impact of STRETCH. The STRETCH team should reduce their meeting frequency to once a month to ensure sustainability in the long-term.<br><br>Share **monthly managers' reports** (data on waiting lists etc).<br><br>Facilitate access to **debriefings by psychologists** through Employee Assistance Programme (EAP). Your PALSA PLUS trainer is also equipped to conduct debriefings.
To ensure a continued supply of the necessary *equipment and supplies*	Assign responsibilities for various equipment and supplies to specific people in the clinic e.g. working fax machine to the admin clerk; drug supplies to the pharmacist/pharmacy assistant.
To manage *staff turnover*	**Orientate and provide training**<br>• Assign one full day to an appropriate clinic staff member to orientate each new staff member.<br>• If new nurse, arrange for provincial ARV training (if appropriate) and inform PALSA PLUS trainer.

Address verticalisation

- Verticalisation of services means clients see several health professionals per visit. This means a longer clinic visit and increased patient load on staff.
- Integration of HIV services into primary care services is a key component of STRETCH. See Decentralisation Checklist on page 21.
- Examine client flow in your clinic: are clerks directing clients to the appropriate nurses?
- Examine drug dispensing in your clinic: can ARVs and other drugs be issued/ dispensed by the same pharmacist/ pharmacy assistant?

Avoiding "peaks" and setting targets

- When ARV initiation becomes available at your clinic, it may be tempting to start many clients on ARVs at once to cut your waiting list.
- This will result in large numbers of clients needing follow-up care all on the same day which may be overwhelming for staff.
- This can be avoided by planning initiation visits carefully:
  - Work out how many clients will need nurse-initiated ARVs each month (we estimate this is around 1/3 of those who qualify each month).
  - Book initiation visits throughout the course of the month (see Batching below for more ideas on planning initiation visits).

Batching

- ARV clients become naturally organised into groups through Drug Readiness Training. Take advantage of this when it comes to initiation and follow-up.
- Advise clients how to take their ARVs in groups (instead of one-on-one) and arrange clinical follow-up on the same day. This saves valuable nurse time and allows clients to develop long-term supportive relationships with others in their group.
- The group approach may not be suitable for clients who are not yet comfortable with disclosure or who wish to discuss sensitive issues in private (e.g. sexual practices, contraception etc.)

Drawing bloods and interpreting results

- Routine HIV care (before and on ARVs) follows a clear course with events at pre-defined periods (e.g. CD4 monitoring).
- It wastes time to see a client once to draw blood for a CD4/viral load/ALT and again to follow-up the result.
- Consider drawing blood at usual check-up and follow-up results the following month (e.g. draw viral load and CD4 at 6 month ARV visit, review results at 7 month visit).
- Alternatively consider pre-filling blood request forms and asking clients to return on an arranged day for only the blood draw, and later for a clinical consultation to discuss the result.
- Ensure a fast-track system for blood draws in is place so that clients don't have to wait long periods simply to have blood taken.

The right person for the right job

- Skilled health workers are a scarce resource. Ensure that your nurses are not doing jobs which could be done by others (e.g. Drug Readiness Training Sessions 1 and 2 by counsellors, admin work by admin clerks and data capturers). Save their valuable clinical skills for clinical work.

Mondays and Fridays

- It is a bad idea to start a new programme (or a new phase of STRETCH) on a day when the clinic is very busy (Mondays) or winding down (Fridays).
- When setting dates for starting different phases of STRETCH, also bear in mind public holidays and other disruptions e.g. polio campaign weeks.

TIPS ON MANAGING STAFF SHORTAGES

A shortage of staff in facilities is a common problem with many causes that results in frustration, low morale and an overwhelming case load. Several approaches may prove helpful depending on the cause in your clinic.

Supporting staff and managing burnout

- A major cause of staff shortages is psychological or physical ill-health. Burnout occurs frequently in clinics with high TB and HIV caseloads.
- Regular support by a middle manager (e.g. ARV co-ordinator, local area manager) comprising frequent visits, empathetic listening and efficient management of problems can engender a feeling of a supportive work environment.
- Consider counselling or psychological support for those staff (as a group or individually) who display signs of burnout. Professional debriefings by a psychologist are available through the Employee Assistance Programme. Your PALSA PLUS trainer is also equipped to conduct debriefings.

Rethinking the "morning clinic" culture

- Clinics tend to be extremely busy in the morning and either empty or close early in the afternoon. Staff are often exhausted by midday.
- Encourage staff to pace their work throughout the working day, taking tea and lunch breaks but working a full day.
- Clients may initially be resistant to this approach but will soon see the benefits of a calmer clinic environment and non-stressed nurses.

Co-ordination of staff leave and attendance at off-site training

- Plan leave together to minimise periods when more than one member of staff is away at the same time.
- Many staff are absent from their facilities as they are attending training workshops elsewhere.
- Plan attendance at off-site training courses and attempt to streamline the training courses attended by nurses.
- Try and avoid repetition of topic in different courses.
- Encourage on-site training.
- Agree on a maximum number of days allowed per nurse per year for attendance at these courses.

Prioritise the filling of vacant posts

- Know how many vacant posts you have at your clinic. This information is readily available from Human Resources.
- Encourage managers to fill vacant posts especially those for which there are usually several applicants e.g. admin clerks, data capturers.
- Spread the word – ask colleagues to keep their ears and eyes open for someone who may be suitable for a position at your clinic.

TASKS AND ROLES: PHC SERVICES NURSE (assessment/combined sites/local clinics)

Phase 1: Site preparation and start decentralisation of HIV care

Clinical responsibilities:
- Suspect and diagnose HIV.
- Stage HIV clients and draw CD4 the ***same day***.
    - CD4 ≤ 200 and/or AIDS ***or*** pregnant with CD4 ≤ 350 and/or AIDS: Refer to ARV nurse (urgently if pregnant, CD4 <50, Kaposi's sarcoma).
    - CD4 ≥ 201 and no AIDS or pregnant with CD4 > 350 and no AIDS: Schedule follow-up visits incl. CD4 counts, arrange PMTCT referral.
- Provide routine HIV care especially: screening for TB, cotrimoxazole prophylaxis to those with Stage 3 or 4 HIV or CD4 Õ200.

Record-keeping:
First visit: ***VOLUNTARY COUNSELLING & TESTING*** or ***HIV FOLLOW-UP: NOT YET ON ARVS.***
Subsequent visits: ***HIV FOLLOW-UP: NOT YET ON ARVS.***

Training:
- PALSA PLUS: ensure you understand how to stage clients, interpret CD4 counts, diagnose TB in HIV positive client, start cotrimoxazole prophylaxis.
- HIV form training: ensure you understand how to complete: ***VOLUNTARY COUNSELLING & TESTING, HIV FOLLOW-UP: NOT YET ON ARVS.***

Phase 2: Decentralisation of HIV care to PHC services nurses and ARV monitoring to ARV nurses

Clinical responsibilities:
- As before.

Record-keeping:
- As before.

Training:
- Attend PALSA PLUS support visits.

Phase 3: Initiation of ARV treatment by nurses in selected cases

Clinical responsibilities:
- As before.

Record-keeping:
- As before.

Training:
- Attend PALSA PLUS support visits.

TASKS AND ROLES: ARV NURSE (assessment/combined sites)

Phase 1: Site preparation and start decentralisation of care

Clinical responsibilities:

- Decide on further management of staged HIV clients:
  - CD4 ≤ 200 and/or AIDS ***or*** pregnant with CD4 ≤ 350 and/or AIDS: Refer to ARV doctor (urgently if pregnant, CD4 <50, Kaposi's sarcoma).
  - CD4 ≥ 201 and no AIDS ***or*** pregnant with CD4 > 350 and no AIDS: Schedule follow-up visits including CD4 counts with the PHC services nurse.
- Re-issue repeat ARVs to clients on treatment, support adherence, monitor side-effects, draw monitoring bloods, book doctor follow-ups.
- Provide routine HIV care especially: screening for TB, cotrimoxazole prophylaxis to those with Stage 3 or 4 HIV or CD4 Õ200.

Record-keeping:

- PHC services nurses now complete *VOLUNTARY COUNSELLING AND TESTING* and *HIV FOLLOW-UP: NOT YET ON ARVS.*
- Complete as required: *HIV FOLLOW-UP: NOT YET ON ARVS, ARV NURSE FOLLOW-UP, REFER TO TREATMENT SITE.*

Training:

- PALSA PLUS: ensure you understand how to interpret ARV monitoring bloods and when to refer ARV clients to a doctor.

Phase 2: Decentralisation of HIV care to PHC services nurses and ARV monitoring to ARV nurses

Clinical responsibilities:

- Manage HIV clients not yet on ARVs as before.
- Continue to re-issue repeat ARVs, support adherence and monitor side-effects.
- Draw and now also interpret monitoring bloods and represcribe ARVs according to PALSA PLUS guidelines (STRETCH edition). Refer ARV clients to the ARV doctor only if problems arise.
- Continue to provide routine HIV care.

Record-keeping:

- Complete these forms as required: *HIV FOLLOW-UP: NOT YET ON ARVS, ARV NURSE FOLLOW-UP, REFER TO TREATMENT SITE.*

Training:

- Continue PALSA PLUS training: ensure you understand when eligible clients are suitable for nurse-managed ARVs, how to work-up clients for ARVs and to initiate treatment.

Phase 3: Initiation of ARV treatment by nurses in selected cases

Clinical responsibilities:

- Evaluate clients eligible for ARVs, and initiate treatment in selected cases according to PALSA PLUS guidelines (STRETCH edition). Refer others to the ARV doctor.
- Continue follow-up of clients on ARVs, including interpretation of bloods and represcription of ARVs. Refer to a doctor only if problems arise.

Record-keeping:

- Complete these forms as required: *ARV BASELINE ASSESSMENT*, *DRUG READINESS TRAINING RECORD (INCL. ARV TREATMENT COMMENCED), ARV NURSE-FOLLOW-UP, REFER TO TREATMENT SITE.*

Training:

- Attend PALSA PLUS support visits in clinic.

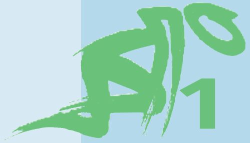

Phase 1: Site preparation and start decentralisation of HIV care

Clinical responsibilities:
- Baseline assessment of eligible patients including exclusion of active TB.
- Initial prescription of ARVs.
- Follow-up of ARV clients (5, 10 and 14 weeks, thereafter 3 monthly) including interpretation of CD4 and viral loads.
- Drug substitution (toxicity) and regimen changes (failure).

Record-keeping:
Unchanged.

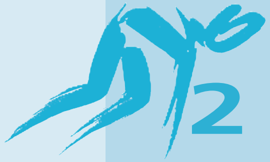

Phase 2: Decentralisation of HIV care to PHC services nurses and ARV monitoring to ARV nurses

Clinical responsibilities:
- Continue baseline assessment of eligible patients and initial ARV prescription.
- Refer all stable ARV clients (from STRETCH clinics only) back to the ARV nurse for further follow-up.
- Continue to monitor all complex cases, and if appropriate substitute drugs or change regimens.

Record-keeping:
- Unchanged.

Training and support:
- Provide support to ARV nurses now assuming responsibility for monitoring of stable ARV clients. Be prepared to accept calls from nurses with queries, and provide feedback on referred cases.

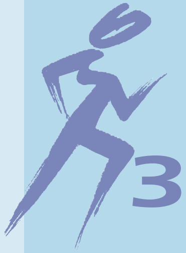

Phase 3: Initiation of ARV treatment by nurses in selected cases

Clinical responsibilities:
- Continue baseline assessment of eligible patients and initial ARV prescription. Attempt to fast-track these cases as only complex or advanced cases are now referred for doctor assessment.
- Refer all stable ARV clients (from STRETCH clinics only) back to the ARV nurse for further follow-up.
- Continue to monitor all complex cases, and if appropriate substitute drugs or change regimens.

Record-keeping:
- As before.

Training and support:
- Provide support to ARV nurses now assuming responsibility for initiation of ARVs in selected clients. Be prepared to accept calls from nurses with queries, and provide feedback on referred cases. Consider visiting the ARV nurse at the clinic to offer support and discuss cases.

TASKS AND ROLES: PHARMACIST/PHARMACY ASSISTANT (treatment sites/assessment sites/combined sites)

Phase 1: Site preparation and start decentralisation of care

At assessment or combined sites:

- Ensure adequate supplies of cotrimoxazole are available.
- Assess storage facilities for ARVs:
  - Is it possible to integrate ARV storage/dispensing/issuing with the usual pharmacy services at the clinic?
  - Would extra shelving in the pharmacy/clinic help? (If yes, contact the provincial STRETCH co-ordinator to arrange.)
- Assess communication systems for Phases 2 and 3. Is a working fax machine in place? If not, contact the facility manager or ARV co-ordinator to arrange.
- Review filing system for ARVs. Suggest filing by ZU number to facilitate quick access when client returns to collect medication.
- Review system for identifying and returning uncollected medication. Are clients' details forwarded to the ARV nurse/community health workers for tracing?
- Review arrangements for transporting ARVs from treatment to assessment sites. How can these be streamlined?

At treatment sites:

- Assess communication systems for Phases 2 and 3. Is a working fax machine in place? If not, contact the facility manager or ARV co-ordinator to arrange.
- Review arrangements for transporting ARVs from treatment to assessment sites. How can these be streamlined?

Phase 2: Decentralisation of HIV care to PHC services nurses and ARV monitoring to ARV nurses

At assessment or combined sites:

- As before. Your will need to accommodate larger numbers of ARVs as clients no longer collect any medication from the treatment site (unless problems arise.)

At treatment sites:

- As before. Once the central pharmaceutical depot for chronic medication, including ARVs, is running, pharmacists at treatment sites will integrate with existing services at that facility.

Phase 3: Initiation of ARV treatment by nurses in selected cases

At assessment or combined sites:

- As before. Storage space for ARVs will need to increase dramatically now as clients start treatment at the clinic, increasing the total number of clients receiving ARVs from that facility.

At treatment sites:

- As before.

Phase 1: Site preparation and start decentralisation of care

HIV clients not yet on ARVs must be seen by PHC services nurses, and no longer the ARV nurse (unless referred).

At assessment/combined sites:

- Direct HIV clients not yet on ARVs to a PHC nurse (with their HIV folder).
- Completion and capturing of forms (and filing of folders afterwards!) must continue for HIV clients who are seen by PHC services nurses.
- Bookings:
  - for HIV clients not yet on ARVs: PHC services nurses, not the ARV nurse (unless referred).
  - for clients on ARVs: ARV nurse or treatment site as usual.
  - for baseline assessments: treatment site as usual.
- Encourage HIV clients to bring their ID books with them so that the ID number can be captured. This is useful for tracking deaths among clients on HIV, which are usually not reported to the clinic. NO ID BOOK DOES NOT MEAN NO CARE. Simply encourage those clients who have them to bring them in at subsequent visits.
- Review filing system:
  - Consider filing by ZU number to enable easy retrieval.
  - Are more filing cabinets needed? If yes, contact the facility manager or ARV co-ordinator to arrange.

At treatment sites:

- Continue as usual.

Phase 2: Decentralisation of HIV care to PHC services nurses and ARV monitoring to ARV nurses

Clients on ARVs will now be mainly followed-up by nurses (STRETCH facilities only) unless problems arise.

At STRETCH assessment/combined sites:

- As before but now book follow-up appointments for ARV clients with the ARV nurse, and not the treatment site unless specifically instructed.

At treatment sites:

- For clients from STRETCH clinics: ask the doctor to indicate clearly whether the next appointment should be at the treatment site or at the STRETCH clinic.
- Note: Clients from non-STRETCH clinics must continue with follow-up as usual (i.e. shared between the assessment and treatment sites).

Phase 3: Initiation of ARV treatment by nurses in selected cases

Selected clients will now be started on ARVs by the ARV nurse at STRETCH assessment/combined sites.

At STRETCH assessment/combined sites:

- Book initiation visits for clients selected for nurse-managed ARVs. Work out the number of clients who can be booked for initiation visits each day with the ARV nurse (see Avoiding "peaks" and setting targets on page 11).

At treatment sites:

- Note that clients referred from STRETCH clinics for baseline assessments are ill or have a co-morbid condition and should be seen by the doctor as soon as possible.

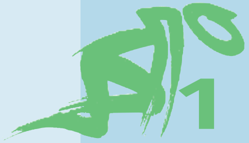

Phase 1: Site preparation and start decentralisation of care

HIV clients not yet on ARVs must be seen by PHC services nurses, and no longer the ARV nurse (unless referred).

At assessment/combined sites:

- Completion and capturing of forms (and filing of folders afterwards!) must continue for HIV clients who are seen by PHC services nurses.
- Ensure adequate supplies of forms are provided to nurses as follows:
  - PHC services nurses: *VOLUNTARY COUNSELLING AND TESTING, HIV FOLLOW-UP: NOT YET ON ARVS*
  - ARV nurses: *VOLUNTARY COUNSELLING AND TESTING, HIV FOLLOW-UP: NOT YET ON ARVS, ARV NURSE FOLLOW-UP*
- ID numbers are useful for tracking deaths among clients on HIV, which are usually not reported to the clinic. Please ensure that they are carefully captured onto MediTech. Note that NO ID BOOK DOES NOT MEAN NO CARE.
- Address problems with MediTech and networks urgently. Don't allow backlogs to grow!

At treatment sites:

- Continue as usual.

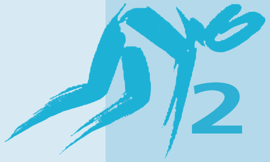

Phase 2: Decentralisation of HIV care to PHC services nurses and ARV monitoring to ARV nurses

Clients on ARVs will now be mainly followed-up by nurses (STRETCH clinics only) unless problems arise.

At STRETCH assessment/combined sites:

- As before but arrange for supplies of *ARV BASELINE ASSESSMENT* forms to be delivered to the ARV nurse in preparation for Phase 3.

At treatment sites:

- As before.

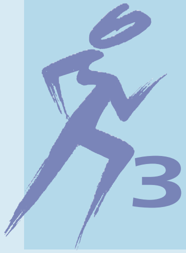

Phase 3: Initiation of ARV treatment by nurses in selected cases

Selected clients will now be started on ARVs by the ARV nurse at STRETCH assessment/combined sites.

At STRETCH assessment/combined sites:

- As before.

At treatment sites:

- As before.

TASKS AND ROLES: PALSA PLUS TRAINER

PALSA PLUS training in STRETCH clinics will differ from usual PALSA PLUS training as follows:

- Extra outreach sessions over a longer time period timed to coincide with critical stages of STRETCH.
- The PALSA PLUS trainer for the STRETCH clinic will be included in that STRETCH clinic support team.
- The training will make use of the special STRETCH edition PALSA PLUS guideline which includes algorithms for classifying eligible HIV clients for nurse- or doctor-managed ARVs, and more detailed recommendations for ARV monitoring.
- The supportive component of the outreach training will be increased.
- Existing PALSA PLUS trainers will be equipped for training in STRETCH clinics during a 2 day STRETCH Training the Trainer to Train (TtTtT) workshop.

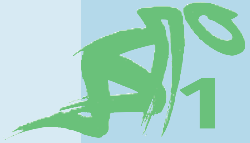

Phase 1: Site preparation and start decentralisation of care

Core knowledge:

- Ensure that all clinic nurses are familiar with the content of the PALSA PLUS guideline and that the management of routine HIV care and TB is embedded in practice. This is needed to facilitate the decentralisation of routine HIV care to PHC services nurses.
- Complete training in new STRETCH edition algorithms: Enrolment in the ARV programme (guideline page 19); Monitoring the client on ARVs (guideline page 21).

Support:

- Participate in the STRETCH clinic support team (see page 6).
- Allocate time during outreach training sessions to the effects of changes brought about by STRETCH: in the facility, in roles, in responsibility.

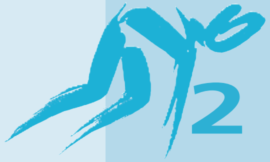

Phase 2: Decentralisation of HIV care to PHC services nurses and ARV monitoring to ARV nurses

Core knowledge:

- As before.

Support:

- Continue involvement in STRETCH support team and PALSA PLUS training of nurses.
- Continue to nurture a relationship of trust with all staff in the clinic.
- Set aside time to be with individual nurses to ensure implementation of Phase 2 changes (see pages 13 and 14).
- Schedule weekly PALSA PLUS contact. Alternate phonecalls with clinic visits.

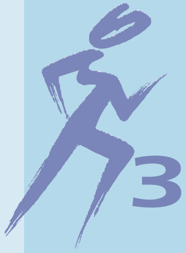

Phase 3: Initiation of ARV treatment by nurses in selected cases

Core knowledge:

- As before. Develop and work through case scenarios (or ask staff to bring client folders to sessions) as this helps to embed knowledge.
- Around 6 to 12 months after initiation review recognition, screening and management of lactic acidosis (guideline pages 22, 25, 32, 34) as this is when clients tend to present with this side-effect.

Support:

- Provide sensitive support around initiation of ARVs.
- If required follow the debriefing process as trained during STRETCH TtTtT: within 12 hours of critical incident or as soon as possible, individually or in a group.

SUGGESTED TIMELINE OF ACTIVITIES

MONTH	WEEK	WEEK STARTING	STRETCH SUPPORT TEAM	PALSA PLUS TRAINING	OTHER
1	1		Team convened	Outreach training	
	2				
	3		Meeting	Outreach training	
	4				
2	5		Meeting	Outreach training	
	6				
	7		Meeting	Outreach training	
	8				Clinic orientation workshop
3	9		Meeting	Outreach training	
	10				Community meeting
	11		Meeting	Outreach training	
	12				Clinic meeting to assess readiness for Phase 2
4	13		Meeting	Outreach training	PHASE 2 STARTS, Brief clients in waiting room
	14		Meeting		Brief clients in waiting room
	15			Outreach training	Brief clients in waiting room
	16		Meeting		Brief clients in waiting room
5	17			Outreach training	
	18		Meeting		
	19			Outreach training	
	20		Meeting		
6	21		Meeting	Outreach training	PHASE 3 STARTS, Brief clients in waiting room
	22		Meeting	Outreach training	Brief clients in waiting room
	23		Meeting	Outreach training	Brief clients in waiting room
	24				

Assess current level of decentalisation of HIV care

Which of the following levels of HIV care are currently handled by Primary Health Care services?

- ☐ Voluntary Counselling and Testing
- ☐ Initial CD4 count
- ☐ Routine HIV care pre-ARVs: serial CD4 monitoring, screening for TB, cotrimoxazole prophylaxis to those with Stage 3 or 4 HIV or CD4 ≤200
- ☐ Drug Readiness Training
- ☐ ARV Baseline bloods
- ☐ Issuing of repeat ARVs

Further decentralisation of HIV care needed at this clinic

Which levels of HIV care *currently* done at the ARV site could be *realistically* decentralised to Primary Health Care services at *your* clinic to enable the ARV sisters to start Phase 2 (monitoring of ARVs) and Phase 3 (initiation of ARVs)?

- ☐ Voluntary Counselling and Testing
- ☐ Initial CD4 count
- ☐ Routine HIV care pre-ARVs: serial CD4 monitoring, screening for TB, cotrimoxazole prophylaxis to those with Stage 3 or 4 HIV or CD4 ≤200
- ☐ Drug Readiness Training
- ☐ ARV Baseline bloods
- ☐ Issuing of repeat ARVs

EQUIPMENT AND SUPPLIES CHECKLIST

Item	Assess	Y/N	Notes
**Waiting room and admissions**			
Waiting room	Does part of the ARV waiting area need to be re-incorporated back into the general waiting room?		
Stigmatising signage	Can any signs directing HIV clients to the ARV area be removed?		
Storage of records	Is it possible for HIV and other records to be stored in a single area? Consider filing HIV records by ZU number to ensure easy retrieval.		
Filing cabinets	Are additional cabinets required?		
**Drug supplies and storage**			
ARVs	Where are these drugs currently stored? Is there sufficient room to accommodate a large increase in supply? Would additional shelving help?		
Cotrimoxazole	Who orders drugs for the clinic? Do they know to expect increased demand for cotrimoxazole?		
**Other supplies**			
EDTA tubes/vacutainer needles	Who orders these? Do they know to order increased quantities?		
Pregnancy tests	Who orders these? Do they know to order increased quantities?		
Sputa jars	Who orders these? Do they know to order increased quantities?		
HIV/ARV forms	Who orders the HIV forms? Do they know to order increased quantities of: VOLUNTARY COUNSELLING AND TESTING, HIV FOLLOW-UP: NOT YET ON ARVS, ARV NURSE FOLLOW-UP and ARV BASELINE ASSESSMENT		
Pap smear equipment	Are speculae available at the clinic? Who orders slides and spatulae? Do they know to order increased quantities?		
**Other**			
Scale	Does the clinic have a scale in working order?		
Laboratory transport	How are bloods transported to the laboratory? By what time should the day's bloods be drawn?		
Laboratory results	How are results returned to the clinic? How are they filed? How do you contact the lab if results are missing?		
Phone	Does the ARV nurse have access to a phone to contact the ARV doctor? Can she/he dial cell numbers?		
Phone access	Does the ARV nurse have sufficient "airtime" to contact the doctor daily? Does this need to be upgraded?		
Fax/ photocopier	Does the clinic have these? Does local area manager know of needs?		
PALSA PLUS guidelines	Does every nurse in the clinic have a PALSA PLUS guideline (STRETCH edition) and materials?		
ARV Treatment guidelines	Does every ARV nurse have a copy of the National Antiretroviral Treatment guidelines (orange book)?		
TB Diagnostic Algorithms	Are these clearly displayed in clinic consulting rooms (not the TB room as this is post-diagnosis!)		

SUGGESTED PROGRAMME FOR ORIENTATION WORKSHOP

Consider holding a half-day orientation workshop in larger STRETCH clinics, about one month before Phase 2 is scheduled to begin.
Scheduling this one mid-week afternoon will limit disruption to clinical services and ensure maximal participation by the clinic.
Ensure the clinic is informed well in advance (3 weeks) so that staff avoid making other commitments for that day.

Who should be invited?
Be inclusive. Many non-health professionals play an important role in the organisation of care at a clinic particularly during staff shortages.
Remember to invite administrative staff, voluntary health workers and cleaners.
Prepare handouts (e.g. STRETCH overview – pages 3 and 4 and relevant task and role sheets – pages 13 to 18).

Introduce STRETCH
Ideally the Facility Manager (STRETCH Team Leader) should facilitate.
Start off by acknowledging that ARVs are working for clients in the Free State, but that too few clients are receiving them.
Highlight the verticalisation of HIV care and the logistical burden this imposes on clients (multiple visits, high transport costs etc.).
Explain what STRETCH stands for.
Describe the step-wise process for introducing STRETCH (see handout).
Explain that phase 1 has already started. Ask about PALSA PLUS training? How is it going? Is it useful?

Allow time for review and discussion
Allow 10 minutes of quiet time (not tea-time) for staff to review handouts. Allow plenty of discussion time.
Review needs equipment and supplies checklist, and actions taken, with clinic staff.
List any additional requirements identified.

Seek buy-in and commitment
Set dates for starting Phases 2 and 3 together. Avoid starting on a Monday or Friday.
Contain anxiety to rush hundreds of clients onto ARVs immediately. Highlight the need for sustainability and avoiding burnout.

Close
Honour the health care workers who have introduced ARVs and comprehensive HIV care under difficult circumstances.
Summarise key activities in Phase 2 and start date.

FREE STATE PROVINCE

Knowledge Translation Unit
University of Cape Town Lung Institute
PO Box 34560
Groote Schuur
7937

Dear Dr Fairall

RE: APPROVAL FOR STRETCH (Streamlining Tasks and Roles to Expand Treatment and Care for HIV) PROJECT

The Free State Department of Health requires that it restructure its Comprehensive Care, Treatment and Management of HIV and AIDS Programme to urgently expand antiretroviral treatment access in the province and reduce waiting times between qualifying for and starting antiretroviral treatment.

STRETCH is a health system intervention which aims to decentralize HIV care, including monitoring of stable patients and initiation of antiretroviral treatment in selected cases, to primary care nurse practitioners without compromising treatment outcomes. Components of the intervention include PALSA PLUS (Practical Approach to Lung Health and HIV/AIDS in South Africa) educational outreach training, a clinical algorithm which stratifies patients as high or low risk and allows nurses to identify patients for nurse- or doctor-managed care, re-defining roles of generalist and ARV nurses and ARV doctors and the establishment of multidisciplinary facility support teams to support intervention facilities.

In order to ensure that the health service offer to patients is not compromised and that the revised clinical roles can be safely and efficiently assumed by health care workers, the STRETCH intervention will first be piloted in 16 of the province's 31 CCMT facilities. The intervention is structured in 3 phases. Phase 1 includes PALSA PLUS training and has already begun as part of provincial implementation of PALSA PLUS; phase 2 involves the decentralization of HIV care to generalist nurses, and ARV monitoring to ARV nurses and is scheduled to begin in July 2007; phase 3 involves the decentralization of ARV initiation to ARV nurses in selected cases and is scheduled to begin in August 2007.

The effectiveness of the STRETCH intervention on programme outcomes will be evaluated by means of a randomized controlled trial commencing in July 2007. After one year, an interim analysis will be completed and the results reported to the Free

Department of Health • Departement van Gesondheid • Lefapha La Bophelo Bo Botle

Acting Head: Health – Dr RD Chapman • PO Box 227, Bloemfontein 9300 • Tel: 051-408 1107
Fax: 051-408 1950 e-mail - chapmard@fshealth.gov.za • 4th Floor, Bophelo House, Cnr. Maitland Street & Harvey Road, Bloemfontein 9300

State Department to decide whether or not to continue the trial, or stop the trial and rollout the intervention or withdraw it.

As accounting officer and acting Head of the Department of Health I grant approval subject to ethics Committee approval for the STRETCH project to proceed as described, including implementation in 16 pilot facilities, and evaluation by means of a randomized controlled trial. Ethics approval can be obtained at any University or Research Organisation.

Yours sincerely

RD Chapman

DR RD CHAPMAN
ACTING HEAD: HEALTH

DATE: ....16/3/07..........

Department of Health • Departement van Gesondheid • Lefapha La Bophelo Bo Botle

Acting Head: Health – Dr RD Chapman • PO Box 227, Bloemfontein 9300 • Tel: 051-408 1107
Fax: 051-408 1950 e-mail - chapmard@fshealth.gov.za • 4th Floor, Bophelo House, Cnr. Maitland Street & Harvey Road, Bloemfontein 9300

ETHICS PERMISSION FOR STRETCH TRIAL EVALUATION

UNIVERSITEIT VAN DIE VRYSTAAT
UNIVERSITY OF THE FREE STATE
YUNIVESITHI YA FREISTATA

Direkteur: Fakulteitsadministrasie / Director: Faculty Administration
Fakulteit Gesondheidswetenskappe / Faculty of Health Sciences

Research Division
Internal Post Box G40
☎(051) 4052812
Fax nr (051) 4444359

E-mail address: gndkhs.md@mail.uovs.ac.za

Ms H Strauss

2007-05-24

DR L FAIRALL
HEAD: KNOWLEDGE TRANSLATION UNIT
UNIVERSITY OF CAPE TOWN LUNG INSTITUTE (PTY) LTD
P O BOX 34560
GROOTE SCHUUR
7937

Dear Dr Fairall

ETOVS NR 75/07
PRINCIPAL INVESTIGATOR: DR L FAIRALL
PROJECT TITLE: A CLUSTER RANDOMIZED CONTROLLED TRIAL OF AN EDUCATIONAL AND ORGANISATIONAL INTERVENTION TO EXPAND ANTIRETROVIRAL TREATMENT ACCESS IN PUBLIC-SECTOR PRIMARY CARE CLINICS IN SOUTH AFRICA: THE STRETCH (STREAMLINING TASKS AND ROLES TO EXPAND TREATMENT AND CARE FOR HIV) TRIAL – STUDY PROTOCOL REC NO IRB00001938.

- You are hereby informed that the following were approved by the Ethics Committee on 22 May 2007 subject to the approval of the Chief Professional Nurses to prescribe HAART:

  - *Detailed Protocol*
  - *Protocol Synopsis*
  - *Information Sheet for Qualitative Evaluation*
  - *Consent Form for Qualitative Evaluation*
  - *Consent form for Economic Evaluation*

- The following documents are used by the Ethics Committee as guidance documents: Declaration of Helsinki, ICH, GCP and MRC guidelines on bio medical research. Clinical trial guidelines 2000 Department of Health RSA; Ethics in Health Research: Principles structure and processes Department of Health RSA 2004, the Constitution of the Ethics Committee of the Faculty of Health Sciences and the guidelines of the S.A. Medicines Control Council as well as laws and regulations with regard to the Control of Medicines.

- Any amendment, extension or other modifications to the protocol must be submitted to the Ethics Committee for approval.

- The Committee must be informed of any serious adverse event and/or termination of the study.

- A progress report should be submitted within one year of approval of longterm studies and a final report at completion of both short term and long term studies.

- Please refer to the ETOVS reference number in correspondence to the Ethics Committee secretariat.

339, Bloemfontein 9300,RSA ☎ (051) 405 2812
Republiek van Suid-Afrika / Republic of South Africa
gndkhs.md@mail.uovs.ac.za

Yours faithfully

for PROF BB HOEK
CHAIR: ETHICS COMMITTEE

PHARMACEUTICAL AND THERAPEUTICS COMMITTEE PERMISSION FOR EXPANDED PRESCRIBING PROVISIONS

MINUTES OF THE PROVINCIAL PHARMACEUTICAL AND THERAPEUTICS COMMITTEE HELD ON 7 MARCH 2007 AT 13H00 IN THE COMMITTEE ROOM (ROOM 3) NATIONAL HOSPITAL

1. **OPENING AND WELCOME**
Dr. Chapman, Acting Head of Health welcomed everyone present. Dr. Chapman apologized to members of the meeting for having to leave at 14h00 because of another important meeting at the Office of the Legislature. Dr. Chapman requested that Mrs Hettie Marais, Manager, Pharmaceutical Services act as chairperson in his absence.
Dr. Chapman thanked all the members of the meeting for their dedication. Dr. Chapman resigned with the Department of Health.
Me Marais and other members wished him well with his new appointment and assured him that he will be sorely missed.

2. **ATTENDANCE AND APOLOGIES**

Attendance

Dr. R.D. Chapman (Chairperson)	Head Office
Me H. M. Marais	Pharmaceutical Services
Me. M. Smith	Pharmaceutical Services
Me. B. Molongoana	RPM Plus
Me. T.P. Oosthuizen	Pharmaceutical Services
Me E.A. Schabort	District pharmacist, DC19
Dr. E.C. Wolmarans	Bethlehem
Dr. H. Dippenaar	Family Medicine
Prof. Odendaal	Dept. Anaesthetics (Pain clinic)
Dr. L. Keet	Dept. Paediatrics
Dr. M. Tshabalala	Treatment of HIV&AIDS Programme
Z. Loots	Pharmacy Universitas Hospital
Prof. Hoek	Department of Paediatrics,Universities
G.J. Kgasane	Pharmacist Pelonomi Hospital
Prof. D.K. Stones	Dept. Paediatrics
Prof. B.W.J. Van Rensburg	Dept. Internal Medicine, Nephrology
F. Gumbi	District Pharmacist, DC16

Apologies
Dr. M. Schoon

3. **CONFIRMATION OF AGENDA**
Agenda approved.

4. **APPROVAL OF MINUTES OF PREVIOUS MEETING**
Approved

5. **MATTERS ARISING FROM PREVIOUS MINUTES**

5.1 **Gadopentetic Acid/Magnevist**
Gadobotrol/Gadovist – Mrs Oosthuizen
The cheaper of the above 2 items can be used in future, depending on the contracts. **LEVEL - 4**

Gadobenic Acid (Gadobenate Dimeglumine = Multihance)
APPROVED – LEVEL 4

5.2 **Raloxifene – Dr. B.J. Van Rensburg**
Dr. Van Rensburg absent from meeting.
Standover for next meeting. (Presenters must be present at meetings to present products.)

5.3 **Teriparatide – Dr. B.J. Van Rensburg**
Dr. Van Rensburg absent from meeting.
Standover for next meeting. (Presenters must be present at meetings to present products.)

5.4 **Calcium Sandoz Eff Tablets**
APPROVED: LEVEL 3 – Tertiary hospital only. (For patients who has difficulty to swallow)

6. **FEEDBACK NEDL**

6.1 Prof Hoek stated that they are busy with the Tertiary list.
6.2 Me Marais stated that she sent a list of all specialist items to NEDLC.
6.3 Dr. Dippenaar stated that they already revised 4 of the Primary Care EDL Book chapters.
6.4 If there is a need for a drug which is included in the new EDL, but which was not previously included in the Free State Code list, a letter, addressed to the PTC is necessary from the relevant department. The letter should state clearly for what indication, the suggested prescribing level as well as the estimated usage.
6.5 If there is a need for another form of a drug which is included in these lists, only a letter is necessary from the relevant department as above.

7. **STRETCH IDEA: NURSE INISIATED ARV TREATMENT – Dr. M. Tshabalala**
The objectives of the presentation were the following:
- To motivate for the provision of antiretrovirals at the 16 STRECH intervention facilities within the context of a rigorous randomized controlled trail evaluation
- To motivate for trained nurse practitioners to initiate first-line antiretroviral treatment in eligible HIV positive adults, provided the conditions listed are met.
- To motivate for trained nurse practitioners to re-prescribe antiretroviral to adults already commenced on treatment provided the indications listed are met.
- To motivate for trained nurse practitioners to provide up to 3 months of ARV's to adults at a time once stabilized on treatment to reduce the burden imposed by follow-up visits to clinics.

The meeting took the following decisions:

Bullet 1: That the normal distribution chain for Hospital level medicines in the province will also be used for antiretrovirals

The Bullets 2 and 3 were approved in principal subject to the following:

(i) Approval has to be obtained from the Ethics Committee to register the program as a trial (research)

(ii) The role of MCC plus the jurisdiction of National Department of Health and allocation of a number will be determined.

(iii) The following protocols must be included in the proposal to the ethics committees:

a) To motivate for trained nurse practitioners to initiate first-line antiretroviral treatment in eligible HIV positive adults provided the conditions listed are met.

b) To motivate for trained nurse practitioners to re-prescribe antiretrovirals to adults already commenced on treatment provided the indications listed are met.

Bullets 4 was not approved.
Nurses cannot dispense the medication as they need to be licensed
Three months medication will not be issued according to a previous decision taken by the PTC on 10 August 2006.

8. NEW MATTERS

8.1 Novo Seven – Dr. Marius Coetzee/Prof Stones
APPROVED – LEVEL 5 (Hematology)ONLY :
Off label emergencies with their approval.
(Universitas Hospital: Prof. Stones & Dr. Marius Coetzee)

8.2 Perfalgan (Paracetamol) IV Injection – Prof Odendaal
APPROVED – LEVEL 3 (ICU & Anaesthesiologists)
Note should be taken that Perfalgan should be infused over no longer than 15 minutes. It is less effective if infused over a longer period. It is safe and effective if infused over less than 15 minutes.
Dosage: Adults and Adolescents
Weight : > 50kg: 1g/administered up to 4x a day.
Minimum intervals between each administration are 4 hours.
Maximum daily dosage is 4 gram.
Prof Odendaal will write protocol

8.3 Losec Mups - Dr. Kriel/Keet
APPROVED – LEVEL 4 (Specialist Peadiatricians)
See pages 39,40,41 and 48 of EDL Paediatrics 2006.

9. CORRESPONDENCE

9.1 Captopril Tablets in the Treatment of Hypertensive Emergencies instead of Nefedipine - Dr. NSC Saidiya
NOT RECOMMENDED
Dr. Dippenaar will discuss the issue at NEDLC meeting and give feedback.

9.2 Melladerm Ointment 20 Gram (An addition) – Prof Sinclair

3 MINUTES OF THE PTC 7 MARCH 2007.doc

CONTACT LISTS

Clinic names and contact details for mangers and co-ordinators at the clinics involved in the trial have been removed to protect their confidentiality.

Compiled by the Knowledge Translation Unit, University of Cape Town Lung Institute
in partnership with the Free State Department of Health.

Department of Health
Departement van Gesondheid
Lefapha La BophelOBo Botle

STRETCH has been developed with the aid of research grants from the
Canadian International Development Agency and Irish Aid.

Irish Aid
Government of Ireland
Rialtas na hÉireann

Canadian International Development Agency
Agence canadienne de développement international

© University of Cape Town.

Disclaimer: This toolkit is the product of ongoing research. While reasonable efforts have been made to ensure the accuracy of the information it contains, the University of Cape Town accepts no liaibility for responsibility for damages or loss arising from the use of this toolkit and/or its contents.
